# Craniofacial tendon development—Characterization of extracellular matrix morphology and spatiotemporal protein distribution

**DOI:** 10.3389/fcell.2022.944126

**Published:** 2022-09-07

**Authors:** Stefanie H. Korntner, Aniket Jana, Elizabeth Kinnard, Emily Leo, Timothy Beane, Xianmu Li, Rohit Sengupta, Lauren Becker, Catherine K. Kuo

**Affiliations:** ^1^ Fischell Department of Bioengineering, University of Maryland, College Park, MD, United States; ^2^ Department of Biomedical Engineering, University of Rochester, Rochester, NY, United States; ^3^ Center for Musculoskeletal Research, University of Rochester Medical Center, Rochester, NY, United States; ^4^ Department of Orthopaedics, University of Maryland Medical Center, Baltimore, MD, United States

**Keywords:** tendon, craniofacial, jaw, tendon development, collagen, lysyl oxidase, matrix metalloproteinase, chick embryo

## Abstract

Craniofacial (CF) tendons are often affected by traumatic injuries and painful disorders that can severely compromise critical jaw functions, such as mastication and talking. Unfortunately, tendons lack the ability to regenerate, and there are no solutions to restore their native properties or function. An understanding of jaw tendon development could inform tendon regeneration strategies to restore jaw function, however CF tendon development has been relatively unexplored. Using the chick embryo, we identified the jaw-closing Tendon of the musculus Adductor Mandibulae Externus (TmAM) and the jaw-opening Tendon of the musculus Depressor Mandibulae (TmDM) that have similar functions to the masticatory tendons in humans. Using histological and immunohistochemical (IHC) analyses, we characterized the TmAM and TmDM on the basis of cell and extracellular matrix (ECM) morphology and spatiotemporal protein distribution from early to late embryonic development. The TmAM and TmDM were detectable as early as embryonic day (d) 9 based on histological staining and tenascin-C (TNC) protein distribution. Collagen content increased and became more organized, cell density decreased, and cell nuclei elongated over time during development in both the TmAM and TmDM. The TmAM and TmDM exhibited similar spatiotemporal patterns for collagen type III (COL3), but differential spatiotemporal patterns for TNC, lysyl oxidase (LOX), and matrix metalloproteinases (MMPs). Our results demonstrate markers that play a role in limb tendon formation are also present in jaw tendons during embryonic development, implicate COL3, TNC, LOX, MMP2, and MMP9 in jaw tendon development, and suggest TmAM and TmDM possess different developmental programs. Taken together, our study suggests the chick embryo may be used as a model with which to study CF tendon extracellular matrix development, the results of which could ultimately inform therapeutic approaches for CF tendon injuries and disorders.

## 1 Introduction

Craniofacial (CF) tendons transfer muscle-generated forces to bones, thus enabling critical jaw movements such as mastication, talking, swallowing, and yawning. Over 3 million CF traumas occur each year in the U.S., involving injuries to the soft tissues and bones of the face and skull, caused by vehicle and sports accidents, assaults, and combat (Miura et al., 2006; Rocchi et al., 2007; Twigg and Wilkie, 2015; Luo et al., 2019; Su et al., 2021). Traumatic CF soft tissue injuries account for nearly 10% of all emergency department visits and up to $200 billion in medical costs (Kretlow et al., 2010; Su et al., 2021). Even though these injuries affect multiple CF tissues, including tendons, current treatment approaches to restore jaw function neglect tendons, and instead focus only on reconstructing bone despite failing to fully restore jaw function (Warren et al., 2003; Zhang and Yelick, 2018). CF tendons also experience painful disorders for which there are no effective treatments. Temporal tendinitis (tendinopathy) is a disorder or disease of the temporalis tendon that has been characterized by both inflammation and degeneration but is under-treated due to the anatomical complexity and incomplete understanding of temporal tendons (Dupont and Brown, 2012; Bressler et al., 2020). Taken together, there is a critical need to understand normal CF tendon formation in order to inform and develop treatment strategies to restore CF tendons and jaw function.

While a small number of studies have investigated CF tendon cell morphogenesis in zebrafish embryos (Chen and Galloway, 2014; McGurk et al., 2017; Subramanian et al., 2018), little is known about CF tendon development. Furthermore, animal models other than zebrafish have not been established for study of CF tendons. In contrast, limb tendon development has been studied in various animal models. We and others have used the chick embryo model to extensively to study limb tendon formation as it closely parallels that in mammals, making it an excellent model to study tendon formation with relevance to human (Fleischmajer et al., 1990; Ros et al., 1995; Birk et al., 1997; Birk and Mayne, 1997; Schweitzer et al., 2001; Edom-Vovard and Duprez, 2004; Canty et al., 2006; Banos et al., 2008; Kuo et al., 2008; Marturano et al., 2013; Marturano et al., 2014; Schiele et al., 2015; Havis et al., 2016; Marturano et al., 2016; Nguyen et al., 2018; Pan et al., 2018; Nguyen et al., 2021; Peterson et al., 2021). Furthermore, the chick embryo has previously been used to study embryonic craniofacial bone, muscle, and cartilage development (reviewed in Abramyan and Richman, 2018). Here, we propose the chick embryo as a model with which to study craniofacial tendon development.

In this study we focused on tendons that have similar jaw-opening and jaw-closing functions as the masticatory tendons in human. It has previously been described in pigeons, parakeets, finches, and tailor birds that the musculus Adductor Mandibulae Externus (mAME) is the principal jaw adductor that enables closing of the lower jaw, and that the musculus Depressor Mandibulae (mDM) is the principal jaw depressor that enables opening and sidewards rotation of the lower jaw (Bhattacharyya, 2013; Carril et al., 2015; Jones et al., 2019; To et al., 2021). We therefore focused on identifying and characterizing the tendons that attach the mAME and the mDM to the lower jaw, which we termed the TmAM (tendon attaching to the mAME) and TmDM (tendon attaching to the mDM). The jaw-closing TmAM and jaw-opening TmDM exhibit similar structure and function as tendons of the human jaw-closing muscles (masseter, temporalis, medial pterygoid) and jaw-opening muscles (lateral pterygoid), respectively (Van Eijden et al., 1997).

During embryonic development, limb tendons undergo drastic changes in cell density and extracellular (ECM) morphology (Kuo et al., 2008; Marturano et al., 2013; Schiele et al., 2015). The ECM of adult tendon is primarily composed of hierarchically organized collagen (Kastelic et al., 1978; Marturano et al., 2013). As the embryonic limb tendon develops, collagen content and organization continue to increase gradually until a functional tendon has formed (Kastelic et al., 1978; Marturano et al., 2013). Collagen type III (COL3) is present at various stages of limb tendon development, and has been implicated in regulating fibrillogenesis of collagen type I as well as fibril growth and assembly during embryonic limb tendon development (Fleischmajer et al., 1988; Fleischmajer et al., 1990; Ros et al., 1995; Birk and Mayne, 1997; Liu et al., 1997; Kuo et al., 2008). Another critical regulator of limb tendon development is lysyl oxidase (LOX), an enzyme that covalently crosslinks collagen to impart mechanical properties to the developing tendon (Marturano et al., 2013; Marturano et al., 2014). LOX mRNA, protein, and LOX-mediated collagen crosslink density increase over time during embryonic limb tendon development (Marturano et al., 2014; Pan et al., 2018). While its function during limb tendon development has been minimally studied, matrix metalloproteinase (MMP)-2 has been detected in chick embryo limb tendons and suggested to play a role in ECM remodeling during development (Jung et al., 2009). Tenascin-C (TNC), a matricellular protein that labels both tendon primordia and differentiated tendons, has been previously used as a marker to label limb tendons during development (Chiquet and Fambrough, 1984; Kardon, 1998; Edom-Vovard et al., 2002). It would be interesting to examine if these regulators and markers of limb tendon development are also present during CF tendon development.

The goal of this study was to characterize the chick embryo as a model with which to study jaw tendon development, with a particular focus on the TmAM and TmDM. We identified the TmAM and TmDM via a combination of functional, anatomical, and morphological features. Using histological and immunohistochemical (IHC) analyses, we characterized the TmAM and TmDM on the basis of cell and ECM morphology and spatiotemporal protein distribution from early to late embryonic development. We focused on ECM molecules implicated in embryonic limb tendon development, and also compared TmAM and TmDM with each other. Our data support the chick embryo as a model to study CF tendon ECM development, and presents morphological and protein characterizations that can serve as a foundation for future studies of CF tendon development.

## 2 Materials and methods

For all experiments chick embryos between developmental day (d) 9 through d19 were used, corresponding to Hamburger and Hamilton (Hamburger and Hamilton, 1951) stages (HH) 35 through HH45, respectively (Table 1). In this study, we refer to the different embryonic stages by developmental days (d). Image analyses of tendon cell density and cell morphology were performed by 3 reviewers in a blinded fashion.

**TABLE 1:** Developmental days (d) and corresponding Hamburger Hamilton stages (HH) of chick embryos used in this study.

Developmental day (d)	Hamburger Hamilton (HH) stage
d9	HH35
d11	HH37
d13	HH39
d15	HH41
d17	HH43
d19	HH45

### 2.1 Gross identification of CF tendon location

Freshly harvested chick embryo heads were used for dissection. After skin and superficial fat were removed, tendons were identified by gently pulling on tendons with forceps to show opening and closing of the beak.

### 2.2 Tissue harvest and processing

Fertilized White Leghorn chicken eggs (CBT Farms, Chestertown, MD) were incubated at 37°C in a humidified rocking incubator. On incubation d9 through d19, chick embryos were sacrificed by decapitation, staged, and heads were fixed in 10% neutral buffered formalin (Sigma-Aldrich, HT501128-4L, MO, United States) overnight at 4°C. After decalcification in Immunocal (StatLab Medical Products, TX, United States) heads were skinned and cut in half along the sagittal plane. Tissue specimens were dehydrated through serially graded ethanol washes, and then processed and embedded in paraffin, as previously described (Kuo et al., 2008; Brown et al., 2012; Navarro et al., 2022). Heads were oriented to section TmAM and TmDM longitudinally at 6 μm thickness. To orient paraffin blocks to obtain longitudinal sections of the TmAM, location and orientation of the eye, ear, and fossa temporalis of the squamosal bone were used as references. To orient paraffin blocks to obtain longitudinal sections of the TmDM, location and orientation of the lower mandible was used as a reference. Tissue sections were subsequently used for histological and immunohistochemical staining.

### 2.3 Histological identification of CF tendons

To identify CF tendons relative to muscle and bone, and visualize tendon morphology, tissue sections were stained with Mallory’s trichrome, as previously described (Kuo et al., 2008; Kuo and Tuan, 2008; Marturano et al., 2013; Navarro et al., 2022), and brightfield images were acquired with a ZEISS Axioscan.Z1 slide scanning microscope with a 20x objective (Zeiss, Oberkochen, Germany).

### 2.4 Characterization of cell density and cell morphology

To assess tendon cell density and morphology, tissue sections were stained with hematoxylin and eosin (H&E), as previously described (Kuo et al., 2008; Kuo and Tuan, 2008; Brown et al., 2012; Marturano et al., 2013; Navarro et al., 2022), and brightfield images were acquired with a ZEISS Axioscan.Z1 slide scanner with a 40x objective. Three non-overlapping same-size (60 × 60 μm) regions of interest (ROIs), equally distributed along the mid-portion of the TmAM and TmDM and avoiding the myotendinous junction and enthesis, were selected for analysis. Brightfield images were deconvoluted into hematoxylin and eosin signals using the H&E macro in Fiji (Schindelin et al., 2012). Cell density was quantified by manually counting nuclei per unit area (mm^2^). Aspect ratio (AR) and circularity of cell nuclei were characterized via image analysis using Fiji. Specifically, nuclei in hematoxylin images were manually selected using the wand tool, after which Fiji would automatically outline the nuclei. Measurements for AR and circularity were then performed automatically by Fiji. Nuclear AR was determined as the ratio of major axis to minor axis. Nuclear circularity was determined as 4 π x (area/perimeter^2^).

### 2.5 Characterization of collagen content, organization, and fiber maturity

To assess collagen content, organization, and maturity, tissue sections were stained with picrosirius red (PSR) and imaged under brightfield and polarized light (POL), as previously described (Kuo and Tuan, 2008), using a ZEISS Axioscan.Z1 slide scanning microscope with a 20x objective. Three non-overlapping same-size (60 × 60 μm) regions of interest (ROIs) were selected equally distributed along the mid-portion of the TmAM and TmDM, avoiding the myotendinous junction and enthesis. Collagen content and organization were analyzed using Fiji. **Collagen content** was characterized by quantifying PSR-stained area in brightfield images. Specifically, images were converted into 8-bit binary images and thresholded to isolate PSR staining from background staining. Percentage area fraction of thresholded PSR staining was calculated. The same threshold was used for all technical and biological replicates for all embryonic stages. **Collagen organization** was characterized by quantifying birefringence signal of POL images, as higher birefringence signal is associated with more organized collagen (Kuo and Tuan, 2008). Specifically, POL images were converted into 8-bit binary images and thresholded to isolate birefringence signal from background. Percentage area fraction of thresholded birefringence signal was calculated. The same threshold was used for all technical and biological replicates for all embryonic stages. **Collagen maturity** was analyzed in POL images of PSR-stained sections using a custom MATLAB routine (version 2019b, Mathworks, Natick, MA). To identify collagen-rich regions of interest, a net intensity threshold was applied to all images as follows: I_R_ + I_G_ + I_B_ ≥ 140, where I_R_, I_G_, and I_B_ represent the red, green, and blue intensity values of individual pixels, respectively. Within the thresholded region, individual pixels were classified as 1) red if I_R_/I_G_ ≥ 1.8; 2) green if I_R_/I_G_ ≤ 1.1; and 3) yellow if 1.1 < I_R_/I_G_ < 1.8. The classification limits were determined by calculating the I_R_/I_G_ intensity ratio of individual pixels for green, yellow, and red. At least 10 pixels were analyzed per embryonic stage for determination of classification limits of each color. Using this classification strategy, area fractions occupied by green, yellow, and red fibers within each ROI were quantified. Relative content of green, yellow, and red regions were interpreted as areas of immature, intermediate, and mature collagen fibers, respectively (Dayan et al., 1989).

### 2.6 Immunohistochemical staining

Immunohistochemical staining was performed to detect TNC, COL3, LOX, MMP2 (gelatinase A), and MMP9 (gelatinase B), based on our previous protocols (Kuo et al., 2008; Brown et al., 2012) and with the following modifications. Briefly, antigen unmasking solution, BLOXALL^®^ endogenous peroxidase blocking solution, normal horse serum (2.5%), ImmPRESS^®^ HRP IgG polymers, and ImmPACT^®^ DAB substrate (Peroxidase, HRP) were purchased from Vector Laboratories (CA, United States). Tissue sections were subjected to a citrate-based heat-mediated antigen retrieval, endogenous peroxidases were blocked for 10 min, and unspecific binding sites were blocked with 2.5% normal horse serum for 1 h. Sections were incubated over night with primary antibodies against TNC (M1-B4, Developmental Studies Hybridoma Bank (DSHB), Iowa, United States, 1:100), COL3 (3B2, DSHB, Iowa, United States, 1:100), LOX (ab31238, Abcam, 1:100), MMP2 (ab97779, Abcam, 1:100), and MMP9 (ab38898, Abcam, 1:100), or with normal horse serum as negative control. Hybridoma Product M1-B4 developed by Fambrough, D.M. and Hybridoma Product 3B2 developed by Mayne, R. were obtained from the DSHB, created by the NICHD of the NIH and maintained at the University of Iowa. After primary antibody incubation, slides were incubated for 1 h with either horse anti-mouse or horse anti-mouse IgG polymer. Subsequently, slides were developed with DAB substrate and counterstained with hematoxylin solution for 30 s (Gill No.1, Sigma, United States). Brightfield images were acquired with a ZEISS Axioscan. Z1 slide scanner with a 40x objective. Using ZEN lite software, three non-overlapping same-size (60 × 60 μm) regions of interest (ROIs) were selected equally distributed along the mid-portion of the TmAM and TmDM, avoiding the attachment zones to the muscle and the bone. Brightfield images were deconvoluted into DAB and hematoxylin signal using the H-DAB macro in Fiji. DAB-images were converted into 8-bit binary images and thresholded to isolate DAB-signal from background. Percentage area fraction of thresholded DAB signal was calculated. For each protein, the same threshold was used for all technical and biological replicates for all embryonic stages.

### 2.7 Statistical analysis

For all image analyses, a minimum of 3 biological replicates (N) were analyzed per embryonic stage. Per biological replicate (N), three ROIs, representing technical replicates (n = 3), were analyzed. Statistical analyses were performed using GraphPad Prism v.8.0.2 (La Jolla, CA, United States). Numerical data is presented as mean ± standard deviation. Each datapoint represents one biological replicate (N), which was determined by average values of 3 technical replicates (n) for that N. For comparison of cell density, cell morphology, collagen content, birefringence, collagen fiber maturity, and IHC-stained area fraction between d9 through d19 jaw tendons, one-way analysis of variance (ANOVA) for multiple comparisons was used followed by a Tukey’s Post hoc test, after confirming normal distribution of the data (D’Agostino and Pearson omnibus normality test). When the above assumption was violated, non-parametric statistics and Kruskal–Wallis test for multiple comparisons was utilized. Statistical significance was set at α = 0.05.

## 3 Results

### 3.1 Gross characterization of jaw-closing TmAM and jaw-opening TmDM

**FIGURE 1:**
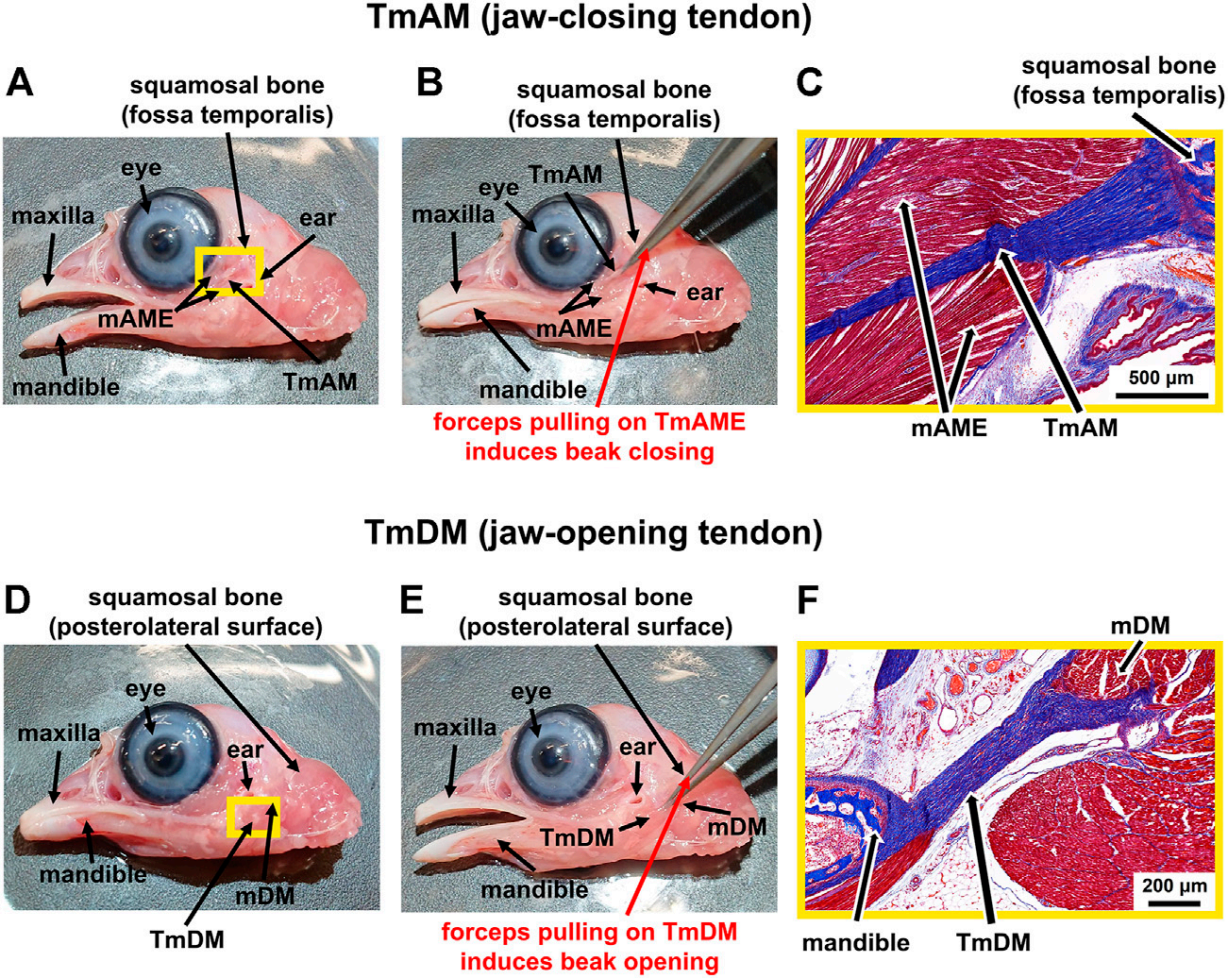
Identification of jaw-closing TmAM and jaw-opening TmDM in a d19 chick embryo. **(A)** Macroscopic view showing location of the jaw-closing TmAM with beak open. **(B)** Pulling the TmAM with a forceps induced closing of the jaw. **(C)** Mallory’s trichrome staining of tissue regions highlighted by yellow rectangle in Figure 1A shows origin of TmAM (blue) at fossa temporalis of squamosal bone (dark blue) and attachment of mAME (red) along both sides of the TmAM. **(D)** Macroscopic view showing location of the jaw-opening TmDM with beak open. **(E)** Pulling the TmDM with a forceps induced opening of the jaw. **(F)** Mallory’s trichrome staining of tissue regions highlighted by yellow rectangle in Figure 1D shows attachments at each end of the TmDM (blue) to mDM (red) and mandible (dark blue).

We grossly detected the TmAM as an intramuscular tendon that originates at the fossa temporalis of the squamosal bone and connects the mAME, a principal jaw adductor muscle, to the lower jaw (mandible) (Figure 1A). The TmAM is involved in rotating as well as lifting the lower jaw upwards and thus is a jaw-closing tendon (Figure 1B). We confirmed the TmAM by using forceps to gently pull on the tendon and showed a closing of the lower jaw (Figure 1B). The TmAM is a pennate tendon with muscle (mAME) attaching along the length of the TmAM on both sides and attachments at the squamosal bone and mandible (Figure 1C). We detected the TmDM as a prominent tendon that connects the mDM, a lower jaw rotator muscle, with the mandible at the posterior end of its medial surface (Figure 1D). The TmDM is involved in rotating the lower jaw as well as protracting the upper jaw (maxilla) and is thus a jaw-opening tendon (Figure 1E). We confirmed the TmDM by using forceps to gently pull on the tendon and demonstrated an opening of the lower jaw (Figure 1E). The TmDM is attached to muscle (mDM) on one end and to bone (mandible) on the other end (Figure 1F).

### 3.2 Histological characterization of jaw-closing TmAM and jaw-opening TmDM

**FIGURE 2:**
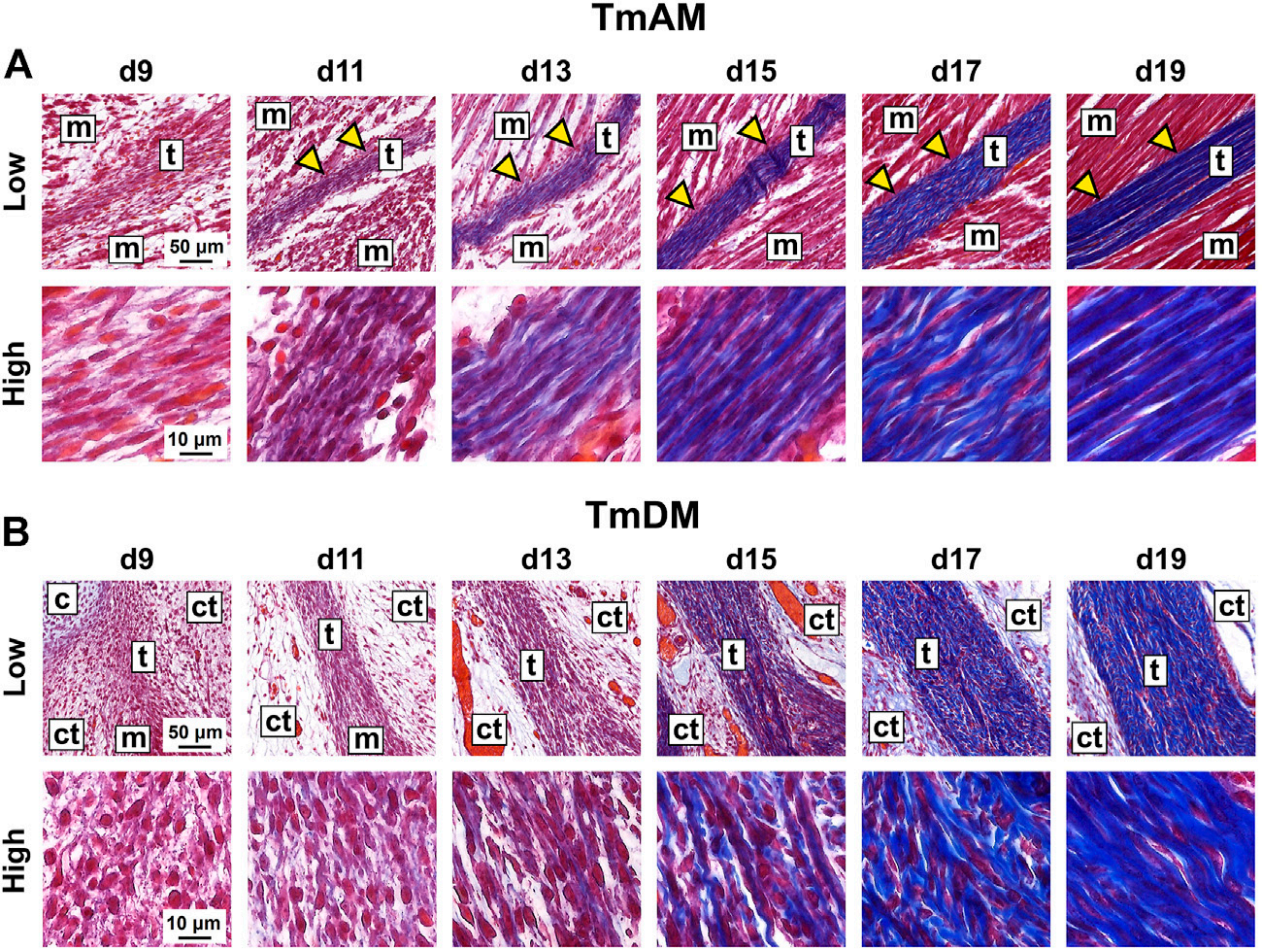
Collagen content appeared to increase and become more organized in jaw-closing TmAM and jaw-opening TmDM between d9 and d19. **(A)** Low and high magnification brightfield images of Mallory’s trichrome staining of TmAM show collagen content (blue) increasing and becoming more organized between d9 and d19. Yellow arrows highlight muscles attaching along length of TmAM. **(B)** Low and high magnification brightfield images of Mallory’s trichrome staining of TmDM show collagen content (blue) increasing and becoming more organized between d9 and d19. Tendon (t), muscle (m), cartilage (c), loose connective tissue (ct).

TmAM and TmDM were first detected by embryonic d9 with histological stains. Tendon structures could not be identified histologically at d8 or earlier (not shown). In trichrome-stained sections, the TmAM was first detected at d9 via blue staining for collagen as well as via attachments to mAME which stained red (Figure 2A). TmAM collagen content appeared to increase and become more organized between d9 and d19 (Figure 2A). Muscle fibers connecting to the TmAM were first detected at d11 (Figure 2A, yellow arrowheads). Similarly, the TmDM was first detected at d9 and distinguishable from surrounding tissue as a denser, more compact microstructure, unlike the surrounding loose connective tissue (Figure 2B). The collagenous matrix of the TmDM stained blue and the attached mDM stained red (Figure 2B). TmDM collagen content appeared to increase and become more organized between d9 and d19 (Figure 2B).

### 3.3 Characterization of tendon cell density and morphology

**FIGURE 3:**
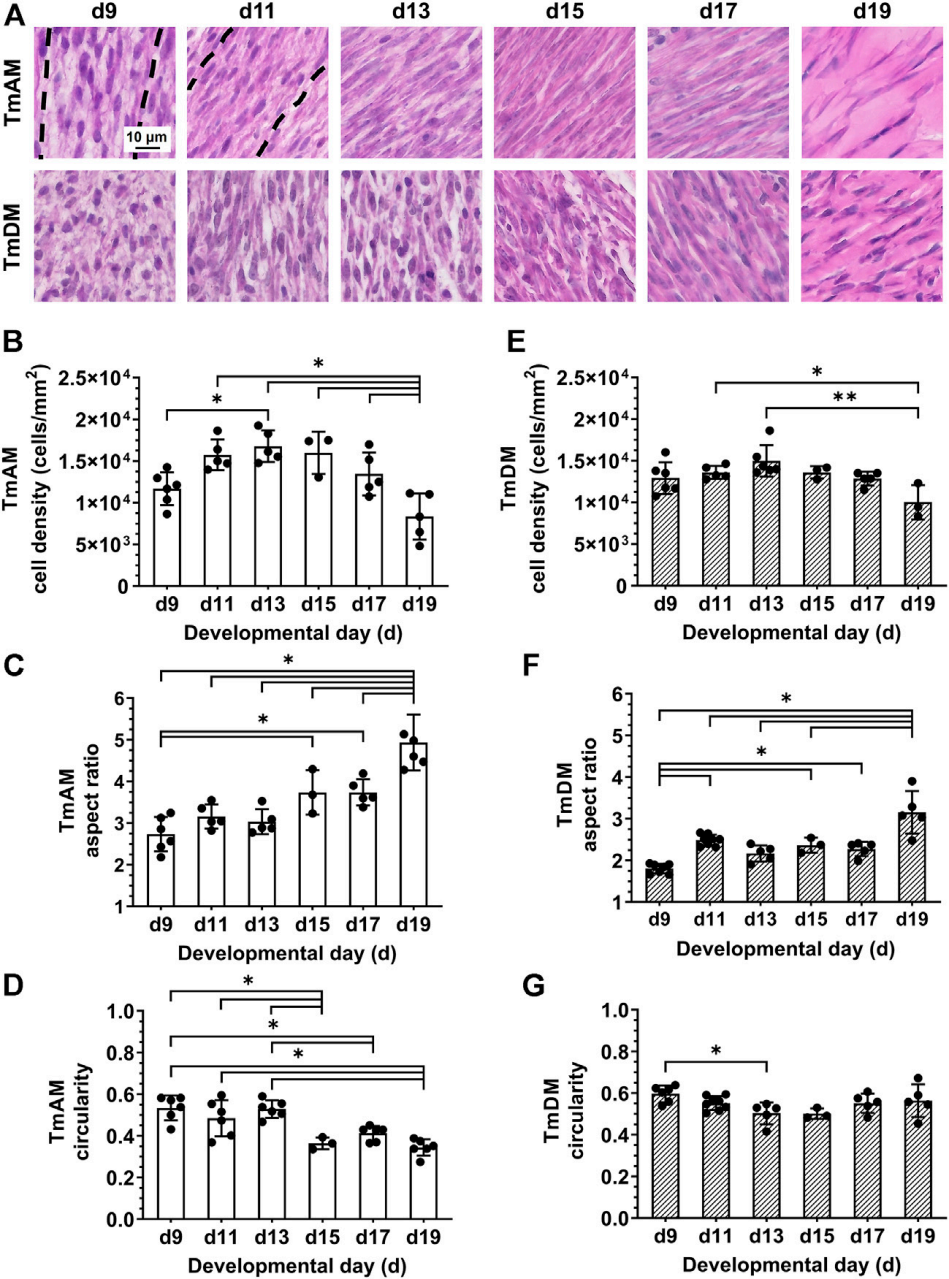
Cell density decreased and nuclei became more elongated in jaw-closing TmAM and jaw-opening TmDM between d9 and d19. **(A)** Representative brightfield images of H&E stained TmAM and TmDM. **(B)** Cell density increased significantly until d13 and subsequently decreased significantly until d19 in TmAM. **(C)** Nuclear aspect ratio increased significantly between d9 and d19 in TmAM. **(D)** Circularity decreased significantly between d9 and d19 in TmAM. **(E)** Cell density decreased significantly between d13 and d19 in TmDM. **(F)** Nuclear aspect ratio increased significantly between d9 and d19 in TmDM. **(G)** Circularity decreased significantly between d9 and d13 in TmDM.

Quantitative image analysis of H&E-stained sections of the TmAM (Figure 3A) revealed a significant increase in cell density between d9 and d13 (Figure 3B). Cell density peaked at d13 in the TmAM and subsequently decreased significantly between d13 and d19 (Figure 3B). Nuclear aspect ratio increased significantly over time during development in the TmAM (Figure 3C). Nuclear circularity remained relatively constant between d9 and d13, decreased significantly after d13, and remained relatively constant between d15 and d19 in the TmAM (Figure 3D). Image analysis of H&E-stained sections of the TmDM (Figure 3A) showed cell density remained relatively constant between d9 and d17, and decreased significantly between d13 and d19 (Figure 3E). Nuclear aspect ratio increased significantly over time during development in the TmDM (Figure 3F). Nuclear circularity decreased significantly between d9 and d13 in the TmDM, and subsequently remained constant between d13 and d19 (Figure 3G). Based on these analyses, tendon cells in both the TmAM and the TmDM changed from a rounded to an elongated shape over time during development.

### 3.4 Characterization of collagen content, organization, and fiber maturity

**FIGURE 4:**
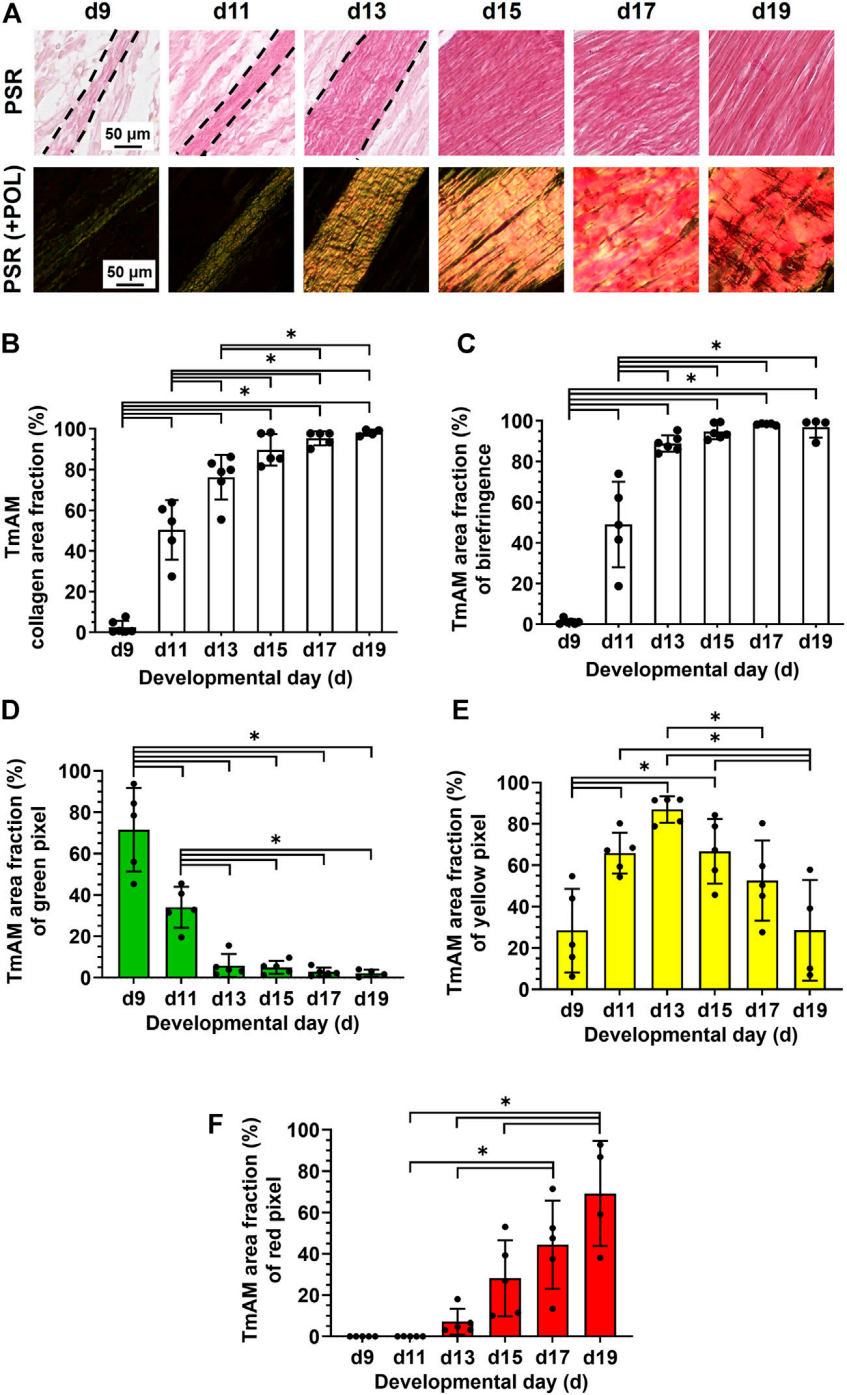
Collagen content increased, collagen became more aligned, and fiber maturity increased in jaw-closing TmAM between d9 and d19. **(A)** Representative brightfield and polarization (POL) images of PSR-stained TmAM. **(B)** Collagen content increased significantly over time and plateaued after d13 in TmAM. **(C)** In the TmAM, collagen birefringence signal increased significantly over time and reached a plateau after d13. **(D–F)** Collagen fibers progressed from mainly immature **(D)** during early stages, to intermediate **(E)**, and finally to mature **(F)** during late stages of development in TmAM.

TmAM collagen content, organization, and fiber maturity were analyzed in PSR-stained sections (Figure 4A). Image analysis revealed collagen content significantly increased from stage to stage between d9 and d13 in the TmAM and reached a plateau after d13 (Figure 4B). Analysis of collagen birefringence signal, a surrogate for collagen organization, revealed significant increases in the TmAM between d9 and d13 (Figure 4C). After d13, collagen birefringence signal reached a plateau in the TmAM (Figure 4C). Based on these data, collagen content increased throughout development in the TmAM, and became more organized during early and intermediate developmental stages. Quantitative analysis of collagen fiber maturity in the TmAM revealed that the percentage of immature (green) fibers decreased significantly between d9 and d13 and remained consistently low after d11 (Figure 4D). Percentage of intermediate (yellow) fibers increased significantly in the TmAM between d9 and d13, peaked at d13, and decreased significantly between d13 and d19 (Figure 4E). Mature (red) fibers were first detected in the TmAM at d11 and percentage of mature fibers increased significantly between d11 and d19 (Figure 4F). Based on these data, collagen fibers in the TmAM progressed from mainly immature during early stages, to intermediate, and finally to mature during late stages of development.

**FIGURE 5:**
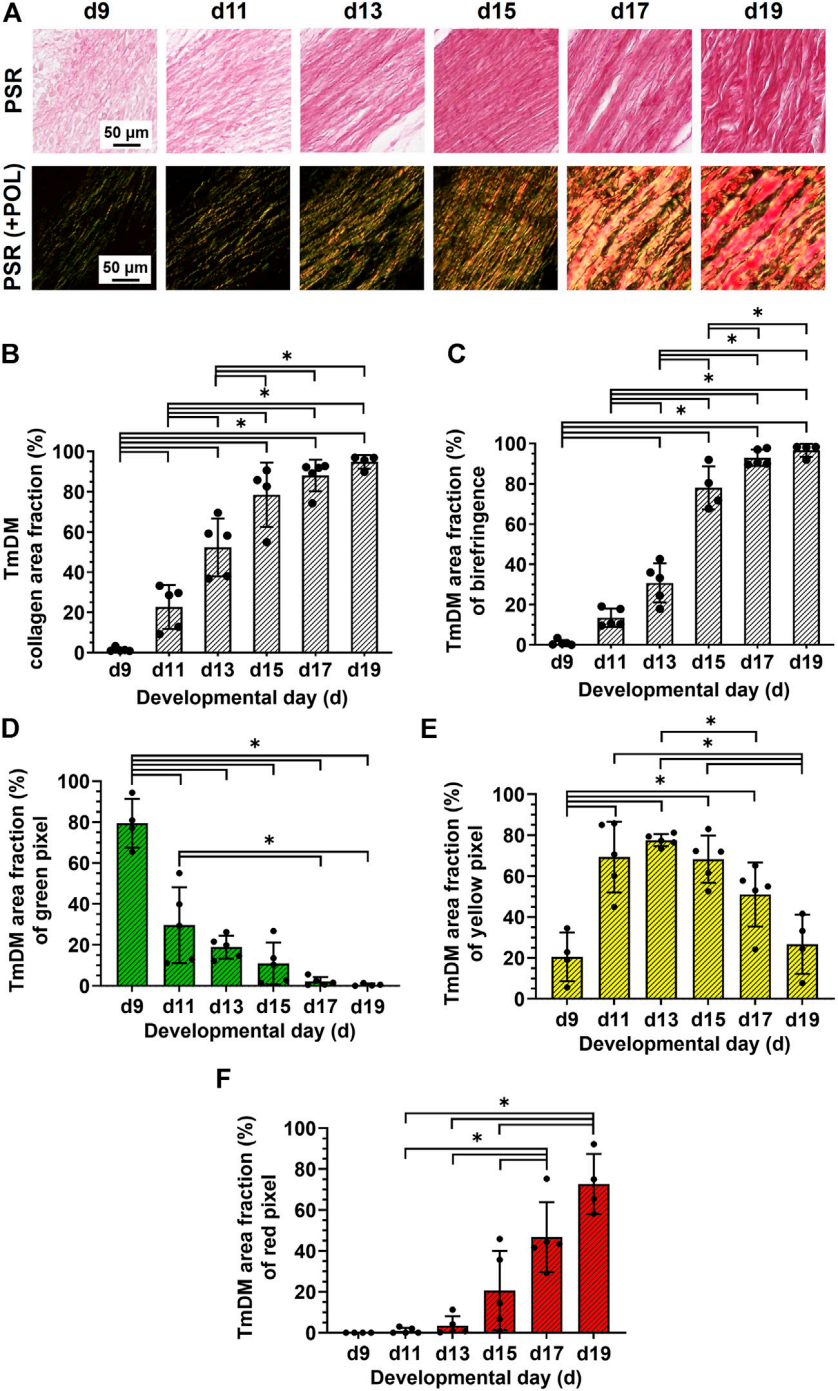
Collagen content increased, collagen became more aligned, and fiber maturity increased in jaw-opening TmDM between d9 and d19. **(A)** Representative brightfield and polarization images of PSR-stained TmDM. **(B)** Collagen content increased significantly over time and plateaued after d15 in TmDM. **(C)** In the TmDM, collagen birefringence increased significantly over time and reached a plateau after d17. **(D–F)** Collagen fibers in TmDM progressed from mainly immature **(D)** during early stages, to intermediate **(E)**, and finally to mature **(F)** during late stages of development.

TmDM collagen content, organization, and fiber maturity were also analyzed in PSR-stained sections (Figure 5A). Image analysis revealed collagen content in the TmDM increased significantly from stage to stage between d9 and d15 and reached a plateau after d15 (Figure 5B). Analysis of collagen birefringence signal revealed significant increases between d9 and d17 and then a plateau after d17 (Figure 5C). Based on this data, collagen content continued to increase in the TmDM throughout development, and became more organized during early and intermediate developmental stages. In the TmDM, percentage of immature (green) fibers decreased significantly between d9 and d11 (Figure 5D). Percentage of intermediate (yellow) fibers increased significantly in the TmDM between d9 and d13, peaked at d13, and subsequently decreased significantly between d13 and d19 (Figure 5E). Mature (red) fibers were first detected in the TmDM at d11 and increased significantly between d11 and d19 (Figure 5F). Based on these data, collagen fibers in the TmDM progressed from mainly immature during early stages, to intermediate, and finally to mature during late stages of development.

### 3.5 Spatiotemporal protein distribution of collagen type III (COL3)

**FIGURE 6:**
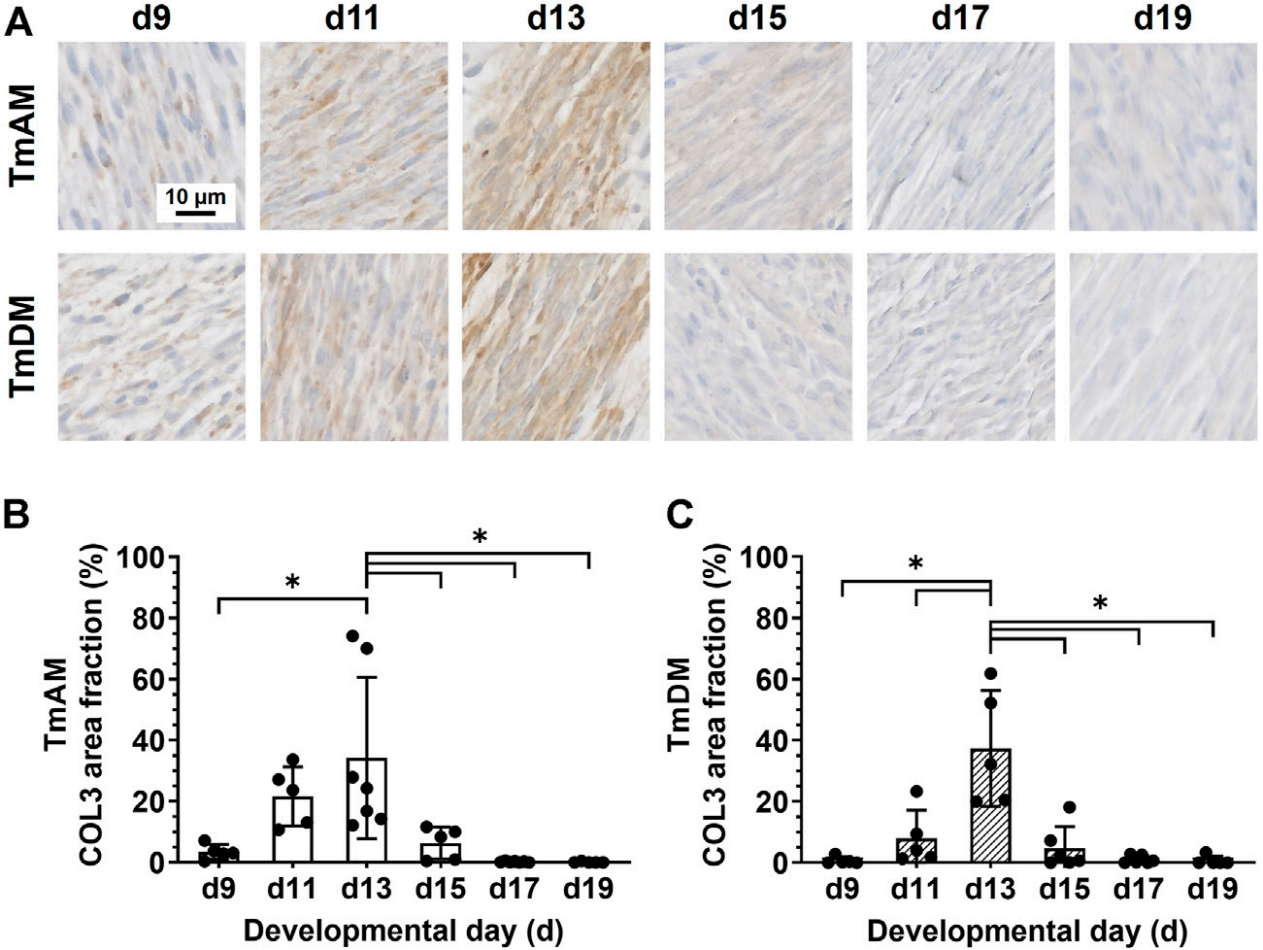
COL3 decreased after d13 in both jaw-closing TmAM and jaw-opening TmDM. **(A)** Representative brightfield images of COL3 IHC of TmAM and TmDM. **(B)** In TmAM, percentage area fraction positive for COL3 increased significantly until d13, and then decreased significantly in TmAM. **(C)** In TmDM, percentage area fraction positive for COL3 increased significantly until d13, and then decreased significantly after d13.

Quantitative analysis of COL3 presence in the TmAM (Figure 6A) revealed that COL3 increased significantly between d9 and d13 (Figure 6B). COL3 peaked at d13 in the TmAM, decreased significantly after d13, and remained low until d19 (Figure 6B). Quantitative analysis of COL3 presence in the TmDM (Figure 6A) revealed that COL3 also increased significantly between d9 and d13 (Figure 6C). COL3 peaked at d13 in the TmDM, decreased significantly between d13 and d15, and remained low during later stages (Figure 6C).

### 3.6 Spatiotemporal protein distribution of tenascin-C (TNC)

**FIGURE 7:**
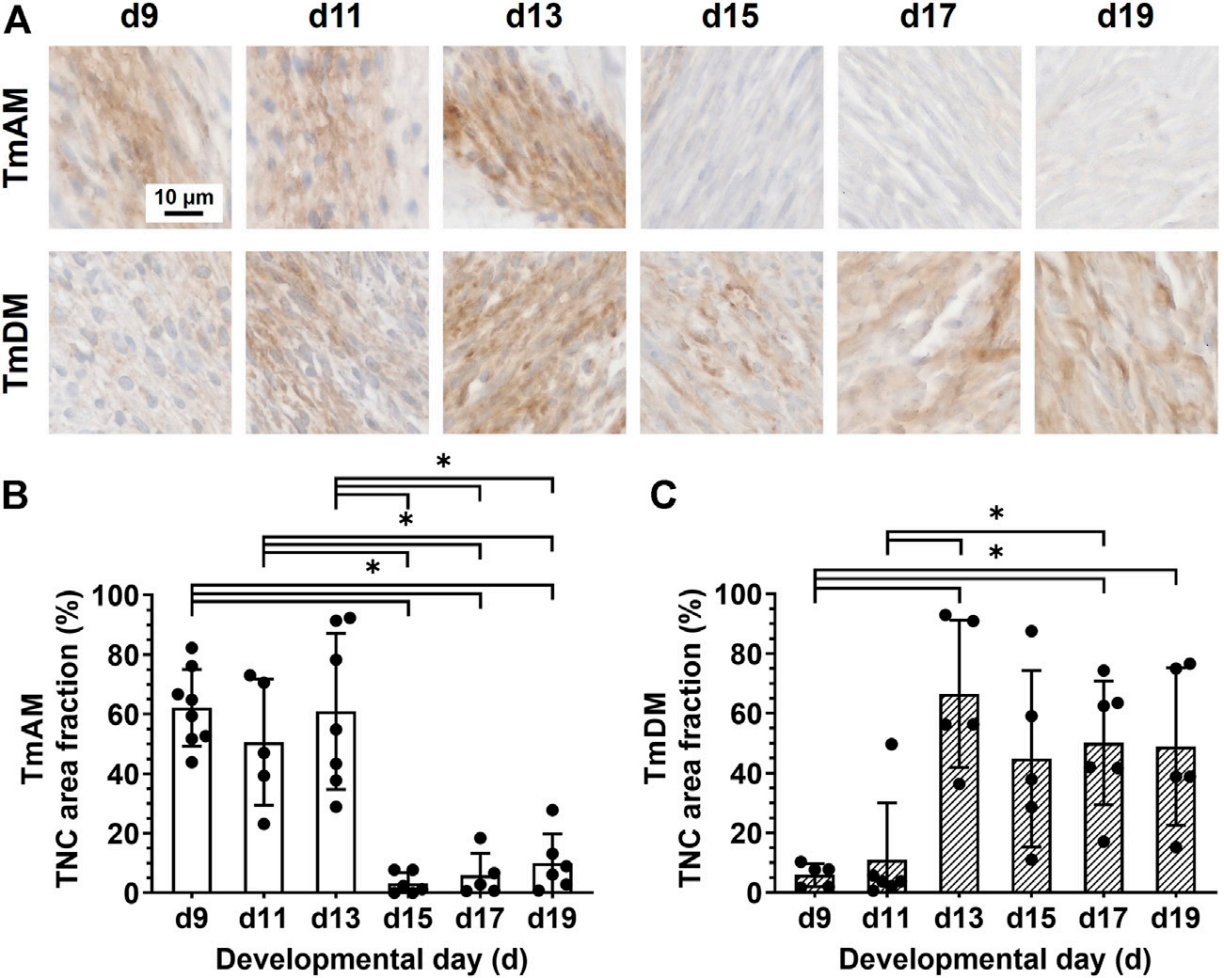
TNC showed different temporal patterns in jaw-closing TmAM and jaw-opening TmDM. **(A)** Representative brightfield images of TNC IHC of TmAM and TmDM. **(B)** In TmAM, percentage area fraction positive for TNC decreased significantly after d13. **(C)** In TmDM, percentage area fraction positive for TNC increased significantly until d13 and then plateaued.

Quantitative analysis of TNC presence in the TmAM (Figure 7A) revealed that TNC remained relatively high between d9 and d13, decreased significantly after d13, and remained low during later stages (Figure 7B). In contrast, quantitative analysis of TNC presence in the TmDM (Figure 7A) revealed that TNC was relatively low at d9 and d11, increased significantly between d9 and d13, and remained relatively constant between d13 and d19 (Figure 7C **)**.

### 3.7 Spatiotemporal protein distribution of lysyl oxidase (LOX)

**FIGURE 8:**
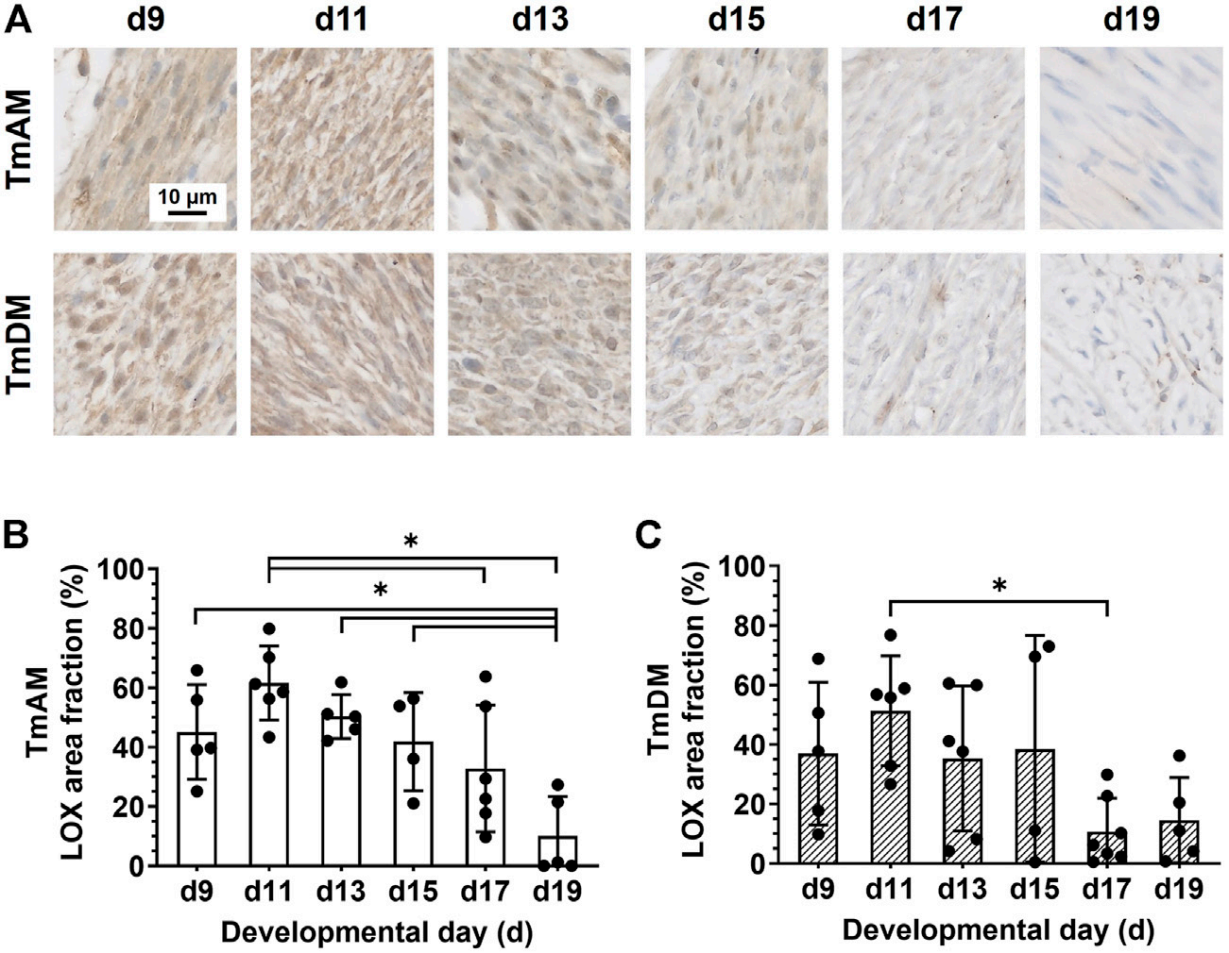
LOX decreased during late developmental stages in jaw-closing TmAM and jaw-opening TmDM. **(A)** Representative brightfield images of LOX IHC of TmAM and TmDM. **(B)** In TmAM, percentage area fraction positive for LOX decreased significantly from d15 to d19. **(C)** In TmDM, percentage area fraction positive for LOX decreased significantly between d11 and d17 in TmDM.

Quantitative analysis of LOX presence in the TmAM (Figure 8A) revealed that LOX was relatively constant during early and intermediate stages, and decreased significantly between d11 and d19 (Figure 8B). Quantitative analysis of LOX presence in the TmDM (Figure 8A) revealed that LOX levels were relatively constant between early and intermediate stages, decreased significantly between d11 and d17, and stayed constantly low until d19 (Figure 8C **)**.

### 3.8 Spatiotemporal protein distribution of matrix metalloproteinase 2 (MMP2)

**FIGURE 9:**
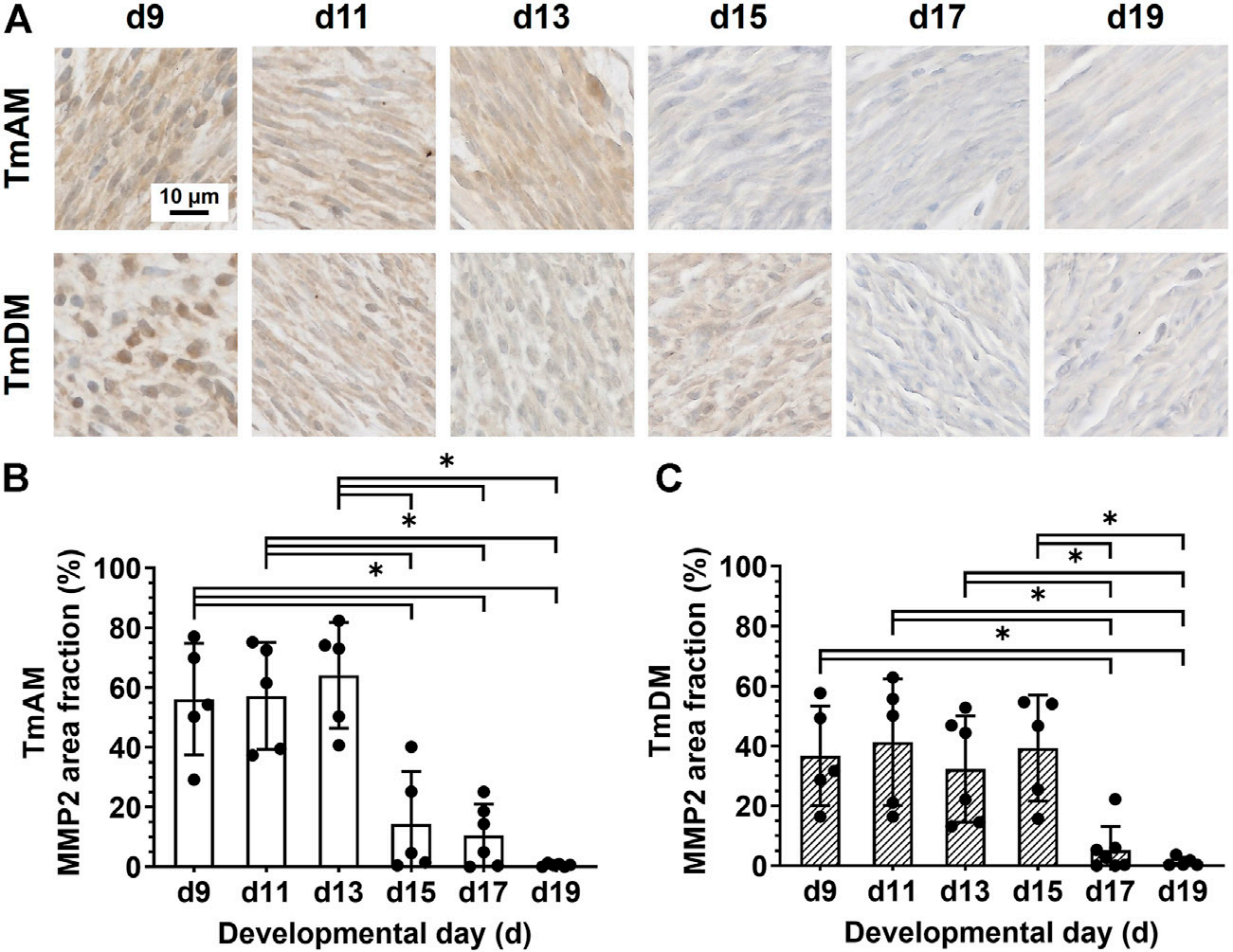
MMP2 decreased during mid-to-later stages in jaw-closing TmAM and jaw-opening TmDM. **(A)** Representative brightfield images of MMP2 IHC of TmAM and TmDM. **(B)** In TmAM, percentage area fraction positive for MMP2 decreased significantly after d13. **(C)** In TmDM, percentage area fraction positive for MMP2 decreased significantly after d15.

Quantitative analysis of MMP2 presence in the TmAM (Figure 9A) showed that MMP2 was relatively constant in the TmAM between d9 and d13, decreased significantly between d13 and d15, and remained constantly low until d19 (Figure 9B). Quantitative analysis of MMP2 presence in the TmDM (Figure 9A) showed that MMP2 remained relatively constant between d9 and d15 (Figure 9C). MMP2 decreased significantly between d15 and d17 and remained low until d19 (Figure 9C).

### 3.9 Spatiotemporal protein distribution of matrix metalloproteinase 9 (MMP9)

**FIGURE 10:**
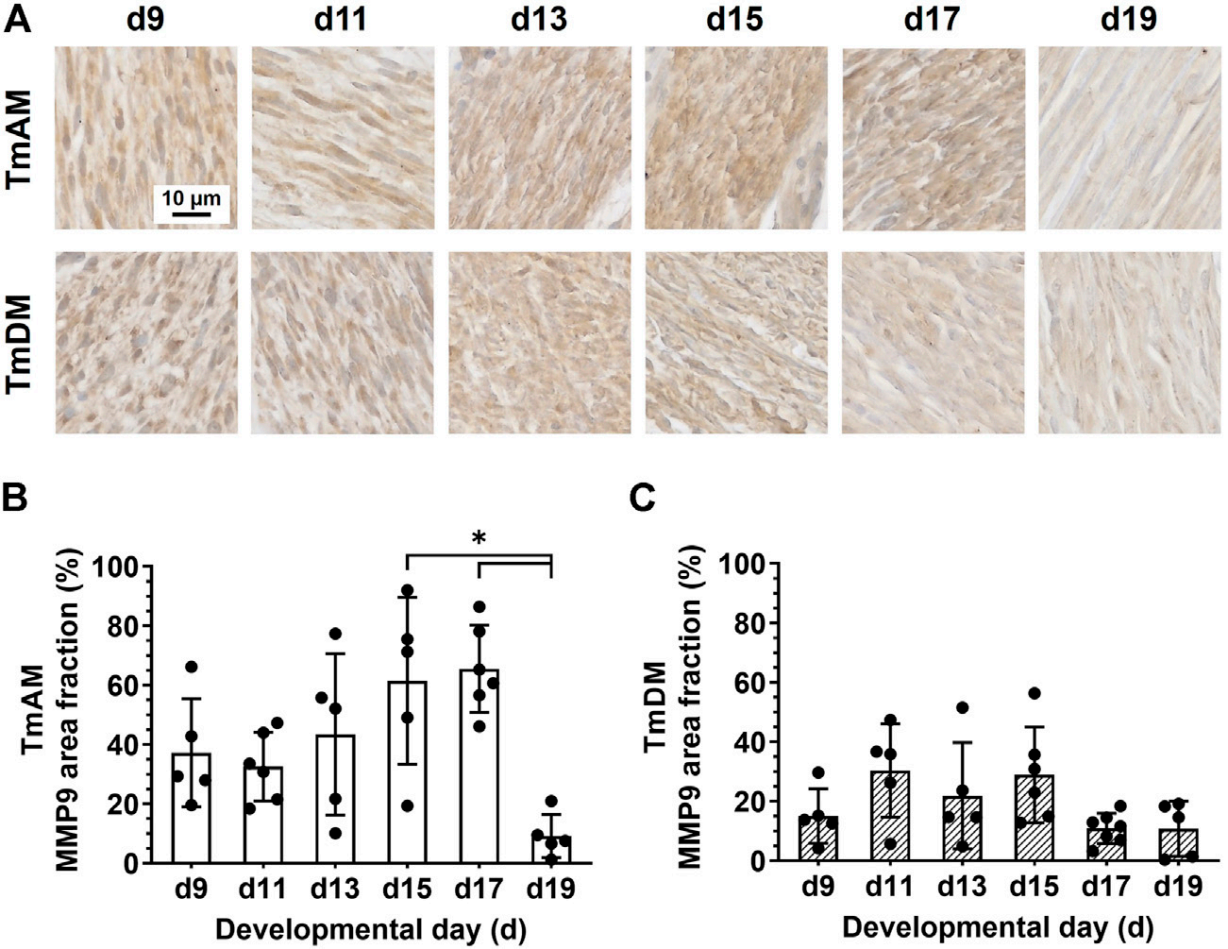
MMP9 was present in jaw-closing TmAM and jaw-opening TmDM between d9 and d19. **(A)** Representative brightfield images of MMP9 IHC of TmAM and TmDM. **(B)** In TmAM, percentage area fraction positive for MMP9 decreased significantly after d17. **(C)** In TmDM, percentage area fraction positive for MMP9 remained relatively constant between d9 and d19.

Quantitative analysis of MMP9 presence in the TmAM (Figure 10A) showed that MMP9 remained relatively constant between d9 and d17 and decreased significantly between d17 and d19 (Figure 10B). Quantitative analysis of MMP9 presence in the TmDM (Figure 10A) showed that MMP9 remained relatively constant between d9 and d19 (Figure 10C).

## 4 Discussion

CF tendon injuries and disorders can severely compromise jaw movements such as mastication, yet there are currently no treatments to regenerate and restore jaw function. Designing treatments for jaw tendon regeneration will require fundamental knowledge about jaw tendon development, which has been minimally studied. Here, we comprehensively characterized spatiotemporal patterns of cell and ECM morphology and protein distribution at distinct embryonic stages to provide markers for TmAM and TmDM development. Our results show markers that play a role in limb tendon formation are also present in jaw tendons during embryonic development, implicate COL3, TNC, LOX, MMP2, and MMP9 in jaw tendon development, and suggest TmAM and TmDM vary in their developmental programs. Our study supports the chick embryo as a novel model with which to study mechanisms of CF tendon development. This model, and the morphological and protein characterizations we present here, will enable future studies that could ultimately inform therapeutic approaches for CF tendon injuries and disorders.

### 4.1 Chick embryos possess jaw-opening and jaw-closing tendons analogous to those in human

The TmAM is a multipennate tendon, as muscle fibers attach along the length of the tendon. Similar to our observations in the chick embryo, tendons of human jaw-closing muscles (masseter, temporalis, medial pterygoid) frequently exhibit a multipennate structure with muscle fibers inserting along the tendon length, which allows for transmission of high forces required for closing of the jaw (Van Eijden et al., 1997). Based on these findings, the TmAM could primarily be responsible for transmission of high forces during jaw-closing. In contrast, the jaw-opening TmDM is a non-pennate tendon, and attaches to muscle at the end of the tendon. Similar to our observations in the chick embryo, tendons of human jaw-opening muscles (lateral pterygoid, digastric, geniohyoid, mylohyoid) are also rarely pennate, and transmit forces to enable excursion and displacement of the jaw during opening (Van Eijden et al., 1997). Based on these findings, the TmDM could primarily be responsible for transmission of forces for excursion and displacement of the lower jaw during opening. These conclusions were corroborated by the jaw movements observed when gently pulling on the tendons (Figure 1).

### 4.2 Jaw tendons are detectable by d9

Both TmAM and TmDM could be first identified at d9, based on collagen presence, TNC labeling, and attachments to muscle and bone or cartilage (Figure 1C,F, Figure 2A,B, Figure 7A). TNC labels early distal tendon primordia between d5 and d7 as well as anatomically distinct tendons arising from these primordia after d7 (Chiquet and Fambrough, 1984; Chiquet-Ehrismann et al., 1986; Hurle et al., 1989; Hurle et al., 1990; Ros et al., 1995; Kardon, 1998). Previous studies by us and others have found that collagen matrix is first detectable via histological staining and second harmonic generation (SHG) imaging in chick embryo limb tendons at d9 (Ros et al., 1995; Marturano et al., 2013). Taken together, our results suggest TmAM and TmDM ECM development begin approximately at the same time as that of limb tendon during embryonic development.

### 4.3 TNC spatiotemporal distribution differs between TmAM and TmDM

TNC exhibited substantially different temporal patterns in the two jaw tendons. In particular, TNC protein distribution in the TmAM was relatively high until d13 and decreased afterwards, whereas in the TmDM it was relatively low during early stages and increased after d11 (Figure 7). TNC has been used as a marker for embryonic limb tendon (Chiquet and Fambrough, 1984; Hurle et al., 1989; Ros et al., 1995; Kardon, 1998; Edom-Vovard et al., 2002). However, even though TNC has been implicated in collagen fibrillogenesis during development of a range of tissues (Mackie, 1994; Riley et al., 1996; Chiquet-Ehrismann and Tucker, 2004), this role has not been definitively shown in tendon. To date, specific TNC functions during embryonic tendon development are largely unknown, yet our data confirms that jaw tendons possess TNC as well.

### 4.4 Jaw tendon cells shift from higher density and rounded morphology to lower density and elongated morphology during development

Cell density appeared constant in both TmAM and TmDM during early embryonic stages and decreased after d13 (Figures 3B,E). Our results here are similar to previously reported declines in tendon cell density during embryonic development based on quantitative DNA assays (Marturano et al., 2013). TmAM and TmDM cells became more elongated during development, reflected by increases in nuclear aspect ratio and decreases in circularity (Figures 3C,D,F,G). Similarly, limb tendon cells have been reported to switch from an initially round to a more elongated shape as embryonic development proceeds (Kannus, 2000). Previous studies have associated CF tendon cell elongation during embryonic development with major mechanical stimulatory events like the onset of muscle contractions in zebrafish (Subramanian et al., 2018). Interestingly, embryonic jaw movements such as “beak clapping”, the rapid opening and closing of the beak, has been observed as early as d9 (Kuo, 1932; Kuo, 1938), and is highest between d14 and d19 (Oppenheim, 1968). Our results on cell elongation in both jaw-opening and jaw-closing tendons demonstrate significant changes around these times (Figures 3C,D,F,G), potentially indicating that alterations in cell shape in the TmAM and TmDM could be possibly driven by beak clapping-induced mechanical stimulation.

### 4.5 Collagen content increases and becomes more organized during development

TmAM and TmDM both increased in collagen content (Figure 4B, Figure 5B), birefringence signal (Figure 4C, Figure 5C), and mature collagen fibers during embryonic development (Figure 4F, Figure 5F). Interestingly, TmAM and TmDM exhibited similar temporal patterns in the shift from predominately immature to intermediate to mature collagen fibers during development (Figures 4D–F, Figure 5D–F). Increases in birefringence during development suggested the ECM of both TmAM and TmDM were increasing in collagen fiber density, thickness, and alignment. Our observations in jaw tendons are consistent with our previous reports using SHG and biochemical analyses, which demonstrated that fibrillar collagen content, density, and alignment increase in limb tendons over time during development (Marturano et al., 2013; Marturano et al., 2014).

### 4.6 COL3 peaks when collagen content and birefringence start to plateau

COL3 is thought to be a critical regulator of collagen fibrillogenesis (Fleischmajer et al., 1988; Fleischmajer et al., 1990; Birk et al., 1997; Liu et al., 1997). COL3 is co-expressed with COL1 in limb tendon fascicles during chick embryonic development and appears to regulate the diameter of COL1 fibrils (Fleischmajer et al., 1988; Fleischmajer et al., 1990; Birk et al., 1997; Liu et al., 1997). Here, COL3 staining distribution increased until d13 in both TmAM and TmDM and decreased during subsequent stages (Figure 6). Similar temporal COL3 patterns have been reported in chick embryo limb tendons by us and others (Birk and Mayne, 1997; Kuo et al., 2008). Before d14, COL3 distribution is detected throughout limb tendon fascicles and is associated with smaller diameter collagen fibrils (Birk and Mayne, 1997; Kuo et al., 2008). After d14, COL3 decreases in limb tendon fascicles and collagen fibril diameter gradually increases (Fleischmajer et al., 1990; Birk et al., 1997; Liu et al., 1997; Kuo et al., 2008). Interestingly, COL3 continues to be detectable and associated with smaller diameter collagen fibrils in the regions surrounding the fascicles (Birk and Mayne, 1997; Kuo et al., 2008). In skin, COL3 deficient mice show abnormal collagen fibril diameter distribution (Liu et al., 1997). Taken together, the presence of COL3 in the TmAM and TmDM may suggest that COL3 contributes to regulation of collagen fibrillogenesis in CF tendons.

### 4.7 Presence of LOX during early and intermediate stages could imply that active collagen crosslinking is occurring

LOX staining was detected in both TmAM and TmDM throughout development and decreased during later stages (Figure 8). We previously showed that LOX activity and LOX-mediated crosslinking play critical roles in regulating the mechanical properties of embryonic limb tendon during development (Marturano et al., 2013; Pan et al., 2018). In particular, inhibition of LOX activity prevented increases in limb tendon elastic modulus by inhibiting the formation of new collagen crosslinks (Marturano et al., 2013). In another study, induction of paralysis during development led to lower LOX activity levels and elastic modulus in limb tendon as compared to controls (Pan et al., 2018). On the other hand, increase in movement frequency led to increases in elastic modulus, whereas inhibition of LOX activity during enhanced movement abrogated the increases in modulus (Pan et al., 2018). Notably, LOX-mediated crosslink density increases during limb tendon development, and these increases correlate with increases in proLOX and LOX activity levels (Marturano et al., 2014; Pan et al., 2018). Based on these data, it is possible that LOX-mediated crosslinking of collagen occurs during early and mid-developmental stages of TmAM and TmDM development. However, because the antibody in this study detects both proLOX and active LOX, future analyses would be needed to confirm formation of LOX-mediated collagen crosslinks.

### 4.8 MMP2 and MMP9 may be involved in tendon development

During embryonic development MMPs are involved in ECM remodeling of various tissues including blood vessels, bone, cartilage, skeletal muscle, lungs, and skin (Birkedal-Hansen et al., 1993; Ravanti and Kähäri, 2000; Page-McCaw et al., 2007; Krane and Inada, 2008), but have been minimally examined in tendon development. Here, we detected MMP2 (Figure 9) and MMP9 (Figure 10) protein in both TmAM and TmDM, suggesting roles in ECM remodeling during development. MMP-2 and MMP-9 show activity against denatured collagen type I and type III molecules and native non-fibrillar collagens (Birkedal-Hansen et al., 1993; Aimes and Quigley, 1995; Allan et al., 1995; Bigg et al., 2007; Baldwin et al., 2019). Additionally, MMP2 cleaves soluble and reconstituted fibrillar collagen type I, whereas MMP9 cleaves soluble collagen types I and III (Aimes and Quigley, 1995; Bigg et al., 2007). MMP2-deficient mice are smaller at birth, have slower growth rate, and exhibit distinct phenotypes of limb, trunk, and head bones, compared to wild-type littermates (Itoh et al., 1997; Inoue et al., 2006). During chick embryonic limb tendon development, MMP2 activity is highest prior to and during collagen fibril growth, implicating a role of MMP2 in fibril growth and matrix assembly (Jung et al., 2009). Similarly, MMP2 was relatively high in TmAM and TmDM during early and mid-developmental stages, and decreased significantly during later stages (Figure 9). Thus, it is possible that MMP2 is involved in remodeling the tendon ECM during early and mid-developmental stages. While little is known about MMP9 in tendon development, MMP9 is expressed at other sites of active tissue remodeling in the developing embryo (Reponen et al., 1994; Alexander et al., 1996), and MMP9 deletion leads to abnormal bone development (Vu et al., 1998). Here, MMP9 levels were relatively high until late developmental stages in TmAM, whereas they remained relatively consistent throughout development in TmDM (Figure 10). Considering the remodeling roles of MMP9 in other embryonic skeletal tissues, presence of MMP9 could indicate stage-specific roles in ECM remodeling in TmAM and TmDM during embryonic development.

### 4.9 TmAM and TmDM may follow different developmental programs

Despite many similarities, the developing TmAM and TmDM also exhibited distinct differences in temporal patterns of specific markers, potentially reflecting different developmental processes. The TmAM reaches a maximum collagen content and birefringence level earlier than TmDM during development, attributed in part to the slightly slower decrease in immature collagen fibers in the TmDM (Figures 4, 5). Differences in cell morphology (Figure 3) could imply differences in developmental processes between the TmAM and TmDM considering regulation of cell shape may be important for limb tendon development (Richardson et al., 2007). In particular, TmAM exhibited greater decreases in nuclear circularity together with greater increases in nuclear aspect ratio over time compared to TmDM, suggesting that TmAM cells elongate to a greater extent compared to TmDM cells. Pennate tendons accumulate muscle forces from varying angles arising from muscle attachments along the length of the tendon. Compared to non-pennate tendons, pennate tendons are functionally stiffer (Farris et al., 2013; Brukner et al., 2018). Based on the differences in how they each attach to muscle, it is likely that the TmAM and TmDM experience different mechanical stimuli during embryonic development. Different mechanical microenvironments experienced by the pennate TmAM and the non-pennate TmDM could be responsible for different temporal patterns in TNC, LOX, and MMP protein distribution (Figures 7–10), as TNC, LOX, MMP2, and MMP9 have each been reported to be regulated by mechanical loading. In particular, TNC expression levels decreased with limb immobilization but increased with treadmill running in Achilles tendons of rats (Järvinen et al., 1999; Järvinen et al., 2003). We have previously shown that paralysis of embryos reduces LOX activity levels in limb tendons compared to controls, suggesting LOX is regulated by mechanical loading of tendon during development (Pan et al., 2018). During late developmental stages, LOX decreases relatively earlier in TmDM compared to TmAM (Figure 8), which may suggest differences in LOX-mediated collagen crosslinking due to differences in mechanical loading of the two jaw tendons. Both MMP2 and MMP9 have been reported to be regulated by mechanical loading in tendon (Koskinen et al., 2004; Huisman et al., 2016). It would be interesting to investigate if earlier decreases in MMP2 (Figure 9) and later decreases in MMP9 (Figure 10) in the TmAM compared to the TmDM are regulated by differences in mechanical loading of the two jaw tendons. The differences in their muscle attachments could also expose TmAM and TmDM differently to muscle-secreted growth factors (Eloy-Trinquet et al., 2009; Wang et al., 2010; Subramanian and Schilling, 2015), which could also have contributed to the differences between TmAM and TmDM outlined above. Taken together, the effects of muscle-induced loading and muscle-secreted factors would be interesting to examine in future studies.

### 4.10 Limitations and future perspectives

The aim of this study was to characterize the chick embryo as a model to study CF tendon formation and to provide distinct morphological and immunohistochemical markers to describe ECM formation of jaw-opening and jaw-closing tendons. To further characterize specific roles of LOX and MMPs in CF tendon development, perturbation of protein and activity levels would be needed. Future studies could also employ additional methods to characterize different aspects of collagen matrix formation in greater detail. The antibodies available for immunohistochemistry at the time of this study could not differentiate between pro-form and active-form of the proteins, and thus future studies should assess LOX and MMP activity levels as well as protein levels of their respective pro-forms.

## 5 Conclusion

This report identified the jaw-closing TmAM and jaw-opening TmDM in the chick embryo that have similar functions as masticatory tendons in human and provided a detailed histological and immunohistochemical characterization of these tissues from early to late embryonic development. Our data implicate TNC, COL3, LOX, MMP2, and MMP9 in CF tendon formation and demonstrated that the two antagonistic tendons develop at different rates with respect to ECM formation and spatiotemporal distribution of tendon-associated and matrix remodeling molecules. Taken together, our study supports the chick embryo as a model with which to study CF tendon ECM development, the results of which could ultimately inform therapeutic approaches for CF tendon injuries and disorders.

## Data Availability

The original contributions presented in the study are included in the article/Supplementary Material, further inquiries can be directed to the corresponding author.
